# COVID-19 #StayAtHome Restrictions and Deep Vein Thrombosis: Case Report

**DOI:** 10.2196/23443

**Published:** 2021-01-14

**Authors:** Edna Blum, Youssef S Abdelwahed, Eileen Spiess, Ursula Mueller-Werdan, David M Leistner, Adrian Rosada

**Affiliations:** 1 Department of Geriatrics Charité Universitätsmedizin Berlin Berlin Germany; 2 Department of Cardiology Charité Universitätsmedizin Berlin Berlin Germany

**Keywords:** thrombosis, public health, social distancing, physical inactivity, pandemic management, COVID-19, case study, vein, adverse effect, physical activity

## Abstract

**Background:**

The COVID-19 pandemic triggered countermeasures like #StayAtHome initiatives, which have changed the whole world. Despite the success of such initiatives in limiting the spread of COVID-19 to #FlattenTheCurve, physicians are now confronted with the adverse effects of the current restrictive pandemic management strategies and social distancing measures.

**Objective:**

We aim to draw attention to the particular importance and magnitude of what may be the adverse effects of COVID-19–related policies.

**Methods:**

We herein report a case of an otherwise healthy 84-year-old woman with deep vein thrombosis (DVT) due to COVID-19–related directives. #StayAtHome policies and consequential social isolation have diminished our patient’s social life and reduced her healthy movement behaviors. The patient spent long hours in a seated position while focusing on the intensive flow of media information regarding the pandemic.

**Results:**

Reduced mobility due to preventive social isolation during the COVID-19 pandemic was the only identified cause of the DVT.

**Conclusions:**

While evaluating the effect of the COVID-19 pandemic and governmentally implemented containment measures, including social isolation and mobility reduction, adverse events should be considered. Digital approaches might play a crucial role in supporting public health.

## Introduction

The COVID-19 pandemic has caused a global state of emergency since the end of January 2020. The spread of this virus has rapidly and strongly affected public, economic, and private life worldwide. Extensive governmental restriction policies were implemented and the media started encouraging social distancing. The catchy phrase #StayAtHome appeared all over the world, reaching everyone through social media, television, banners, and newspapers. These strict measures in the fight against COVID-19 have led not only to social isolation, but also to enhanced restricted mobility in vulnerable groups [1,2]. This case report aims to highlight the importance of implementing preventive measures (eg, via digital approaches) to protect public health during this pandemic.

## Methods

An otherwise completely healthy 84-year-old woman was admitted to the hospital due to deep vein thrombosis (DVT) in mid-April 2020. She had no pre-existing conditions, she was not taking any medication, and she did not have any cardiovascular risk factors apart from her age. Before the COVID-19 pandemic, the patient was very mobile for her age; she regularly went out for shopping and walks with her husband, and was active in her church group. The patient decided to reduce her social activities as a consequence of the increasing flow of alarming information on COVID-19 infections already being reported at the end of January 2020. The appearance of the slogan #StayAtHome and consequent social isolation, along with a later-implemented governmental contact ban, have extremely diminished her social life and everyday activities. Several hours each day were spent sitting in front of the television, extensively focusing on the overwhelming flow of media information concerning COVID-19, as well as new containment measures and the pandemic’s effects on society and the economy. Due to her age and daily media information consumption, she developed anxiety about leaving her home and did not leave her apartment for 6 weeks prior to hospitalization.

Approximately 2-3 days prior to hospitalization, the patient had increasing pain and tenderness, as well as redness and swelling, in her left leg.

On the day of hospitalization, the patient called her primary care physician, who advised her not to come to his medical practice but to start immediate oral anticoagulation therapy with Dabigatran since the patient’s husband was already on this medication for atrial fibrillation. Furthermore, he referred her to the emergency department.

## Results

On admission, the patient presented with redness, increasing calf temperature, and positive thrombosis signs (Wells score of 4 points). No injury or exceptional muscle use in the days before the initial pain were reported. There was no clinical evidence for hemodynamic instability or pulmonary embolism, and the patient had no history of prior thromboembolic events. Anamnestic, clinical, and diagnostical evaluation of the etiology of the DVT took place and showed no evidence of any other cause than reduced mobility due to preventive COVID-19 isolation.

**Figure 1:**
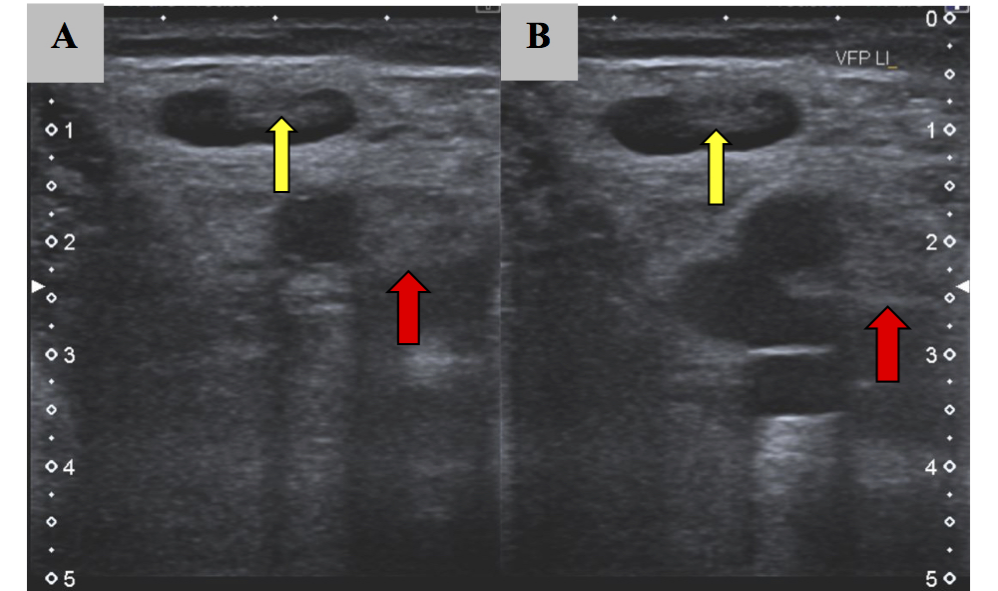
Ultrasound of the left lower extremity showing noncompression of the left saphenous vein (red arrows) and left posterior tibial vein (yellow arrows), as well as a visible thrombus in the lumen. Panel A and Panel B show a well-formed Thrombus in two different locations.

Blood tests revealed leukocytosis (12.94 × 10^9^/L), increased C-reactive protein (13.3 mg/L), and abnormal D-Dimer concentrations (2.38 mg/L). Doppler sonography showed a long-distance thrombosis in the small saphenous vein and posterior tibial veins in her left leg (Figure 1).

The patient was diagnosed with provoked DVT, and it was recommended that she continue the anticoagulation treatment with Dabigatran for 3 months. A 2-week hospitalization became necessary as the patient’s mobility was impaired by the DVT. Physical rehabilitation was initiated and following physiotherapeutic treatment, the patient’s mobility markedly improved. The patient was informed about the most likely cause of her condition and instructed to maintain her physical activity at home by using the stairs on a daily basis and taking walks in the neighborhood while adhering to protection rules.

## Discussion

To our knowledge, we herein report for the first time a case of DVT as a result of #StayAtHome directives and preventive social isolation during the COVID-19 pandemic in octogenarians. No other cause than the reduction of physical mobility due to preventive social isolation was identified.

Restrictive policies including social distancing have been implemented by the government, with the aim of reducing the transmission of COVID-19 and keeping the mortality rate low [3]. However, since this is the first larger worldwide pandemic in the last 100 years [4,5], it is crucial to take into consideration that #StayAtHome directives themselves can have adverse effects, such as a reduction in physical activity and the accompanying consequences. These include not only physical issues (eg, an increased risk of cardiovascular diseases and thromboembolism), but also psychological effects and the impairment of public well-being [6-8].

As illustrated by this case, adverse patient outcomes like DVT can be traced directly to the effects of #StayAtHome directives [9]. Next to seated immobility in relation to travel, work, or computer gaming, a sedentary lifestyle due to COVID-19 restrictions could present as another cause of seated immobility thromboembolism (SIT) [9,10]. Therefore, effective prevention and protection concepts are indispensable during the COVID-19 pandemic, especially for vulnerable and older patients [11-13].

The COVID-19 pandemic has already lasted for several months, adverse effects have already been discussed in scientific forums, and nonpublic institutions have implemented prevention programs (eg, home workout instructions), which have shown that adequate prevention programs are possible while adhering to transmission reduction rules. Nevertheless, governmental policies still lack sufficient consideration of how to integrate measures for adopting positive health-related behaviors into the pandemic management strategy [14]. New strategies including population-wide thrombosis prophylaxis performed at home (such as practicable mobility programs on public television or the enhanced use of digital preventive medicine) should be developed, with special attention paid to the older adult population. Teleworking and teleschooling are already playing a crucial role in this pandemic situation, while integration of telehealth into public health management is, generally speaking, overdue [15,16]. Digital health apps could play an essential role for both psychological issues and physical conditioning [17]. In addition, quality social interactions using telecommunication should be supported since they are negatively associated with the deterioration of physical and psychological health [18]. Moreover, it is important to develop simple and safe solutions (eg, via public television, radio, telephone, newspapers, or flyers) for older people who are not confident using new digital technologies [11].

During this crisis, the implementation of digital approaches to protect public health and avoid an increased mortality rate due to pandemic-related measures is of particular importance [19,20]. We suggest that instead of #StayAtHome, the message could be #StayHomeKeepMoving.

## Abbreviations

DVT: deep vein thrombosis
SIT: seated immobility thromboembolism

## References

[1] Markowitz J (2020). Virtual treatment and social distancing. The Lancet Psychiatry.

[2] Guan H, Okely AD, Aguilar-Farias N, del Pozo Cruz B, Draper CE, El Hamdouchi A, Florindo AA, Jáuregui A, Katzmarzyk PT, Kontsevaya A, Löf M, Park W, Reilly JJ, Sharma D, Tremblay MS, Veldman SLC (2020). Promoting healthy movement behaviours among children during the COVID-19 pandemic. The Lancet Child & Adolescent Health.

[3] World Health Organization (2020). Coronavirus disease 2019 (COVID-19) - Situation report 72.

[4] Martini M, Gazzaniga V, Bragazzi NL, Barberis I (2019). The Spanish Influenza Pandemic: a lesson from history 100 years after 1918. J Prev Med Hyg.

[5] Naguib MM, Ellström P, Järhult JD, Lundkvist Å, Olsen B (2020). Towards pandemic preparedness beyond COVID-19. Lancet Microbe.

[6] Maugeri G, Castrogiovanni P, Battaglia G, Pippi R, D'Agata V, Palma A, Di Rosa M, Musumeci G (2020). The impact of physical activity on psychological health during Covid-19 pandemic in Italy. Heliyon.

[7] Peçanha T, Goessler KF, Roschel H, Gualano B (2020). Social isolation during the COVID-19 pandemic can increase physical inactivity and the global burden of cardiovascular disease. Am J Physiol Heart Circ Physiol.

[8] Zvolensky MJ, Garey L, Rogers AH, Schmidt NB, Vujanovic AA, Storch EA, Buckner JD, Paulus DJ, Alfano C, Smits JAJ, O'Cleirigh C (2020). Psychological, addictive, and health behavior implications of the COVID-19 pandemic. Behav Res Ther.

[9] Ali A, Omore I, Asare L, Gabani M, Riaz M (2020). Seated-immobility thromboembolism syndrome complicating coronavirus disease 2019 outbreak quarantine. Chest.

[10] Healy B, Levin E, Perrin K, Weatherall M, Beasley R (2010). Prolonged work- and computer-related seated immobility and risk of venous thromboembolism. J R Soc Med.

[11] Goethals L, Barth N, Guyot J, Hupin D, Celarier T, Bongue B (2020). Impact of Home Quarantine on Physical Activity Among Older Adults Living at Home During the COVID-19 Pandemic: Qualitative Interview Study. JMIR Aging.

[12] Jones DS (2020). History in a Crisis — Lessons for Covid-19. N Engl J Med.

[13] Parmet WE, Sinha MS (2020). Covid-19 — The Law and Limits of Quarantine. N Engl J Med.

[14] Merkel A (2020). Introductory statement by the Federal Chancellor at the press conference after the Conference of Minister-Presidents (MPK).

[15] Schulman KA, Richman BD (2019). Toward an Effective Innovation Agenda. N Engl J Med.

[16] Tuckson RV, Edmunds M, Hodgkins ML (2017). Telehealth. N Engl J Med.

[17] Pérez Sust P, Solans O, Fajardo JC, Medina Peralta M, Rodenas P, Gabaldà J, Garcia Eroles L, Comella A, Velasco Muñoz C, Sallent Ribes J, Roma Monfa R, Piera-Jimenez J (2020). Turning the Crisis Into an Opportunity: Digital Health Strategies Deployed During the COVID-19 Outbreak. JMIR Public Health Surveill.

[18] Wang P, Ko N, Chang Y, Wu C, Lu W, Yen C (2020). Subjective Deterioration of Physical and Psychological Health during the COVID-19 Pandemic in Taiwan: Their Association with the Adoption of Protective Behaviors and Mental Health Problems. Int J Environ Res Public Health.

[19] Bachireddy C, Chen C, Dar M (2020). Securing the Safety Net and Protecting Public Health During a Pandemic: Medicaid's Response to COVID-19. JAMA.

[20] Inglesby TV (2020). Public Health Measures and the Reproduction Number of SARS-CoV-2. JAMA.

